# Variability in venom composition of European viper subspecies limits the cross-effectiveness of antivenoms

**DOI:** 10.1038/s41598-018-28135-0

**Published:** 2018-06-29

**Authors:** Giulia Zanetti, Elisa Duregotti, Carlo Alessandro Locatelli, Andrea Giampreti, Davide Lonati, Ornella Rossetto, Marco Pirazzini

**Affiliations:** 1 University of Padova, Department of Biomedical Sciences, Padova, 35131 Italy; 2 Istituti Clinici Scientifici Maugeri, IRCCS Maugeri Hospital and University of Pavia, Poison Control Centre and National Toxicology Information Centre - Toxicology Unit, Pavia, 27100 Italy; 3 Present Address: King’s College London, Department of Cardiology, James Black Centre, London, SE5 9NU United Kingdom

## Abstract

Medically relevant cases of snakebite in Europe are predominately caused by European vipers of the genus *Vipera*. Systemic envenoming by European vipers can cause severe pathology in humans and different clinical manifestations are associated with different members of this genus. The most representative vipers in Europe are *V*. *aspis* and *V*. *berus* and neurological symptoms have been reported in humans envenomed by the former but not by the latter species. In this study we determined the toxicological profile of *V*. *aspis* and *V*. *berus* venoms *in vivo* in mice and we tested the effectiveness of two antivenoms, commonly used as antidotes, in counteracting the specific activities of the two venoms. We found that *V*. *aspis*, but not *V*. *berus*, is neurotoxic and that this effect is due to the degeneration of peripheral nerve terminals at the NMJ and is not neutralized by the two tested antisera. Differently, *V*. *berus* causes a haemorrhagic effect, which is efficiently contrasted by the same antivenoms. These results indicate that the effectiveness of different antisera is strongly influenced by the variable composition of the venoms and reinforce the arguments supporting the use polyvalent antivenoms.

## Introduction

Each year, hundreds of thousands of individuals worldwide are affected by snakebite envenomation. This phenomenon represents a public health problem especially in tropical and sub-tropical areas, endemic for the most venomous snake species. Despite the huge payload in terms of human deaths and permanent disabilities or mutilations, this issue is still largely neglected^1,2^. Snakebites are relatively uncommon in Europe, yet the presence of some snakes of the genus *Vipera* accounts for serious envenomation possibly linked to long term sequelae or even death. *V*. *berus*, *V*. *aspis* and *V*. *ammodytes* are the most dangerous species and those responsible for the most severe cases of snakebite envenomation^3^. *V*. *berus* is the most widespread species, present both in north-east and central Europe, including the UK and the north of France and Italy, whereas *V*. *ammodytes* populates a much smaller territory, almost exclusively restricted to the Balkans. *V*. *aspis* is instead indigenous of central and southern France, Swiss and Italy where it shows in some areas an overlapping distribution with *V*. *berus*^3,4^.

Snakebites from *Vipera* snakes cause mainly local effects like pain, edema, swelling and possibly local necrosis. However, in most severe cases, especially in children, local effects may be very serious and systemic symptoms like gastrointestinal issues, hypotension, coagulopathy and neurotoxicity can occur as well. Neurotoxic manifestations mainly affect cranial nerves, leading to botulinum-like symptoms as ptosis, ophthalmoplegia, diplopia, dysphonia, paresthesia, dyspnea and deficit of masticatory, sternocleidomastoid, and nuchal muscles^5^. Treatment is based on hospitalization (if necessary) and on antivenom administration to prevent clinical worsening of envenomation, thus reducing long-term effects and the hospital stay. Available antisera are generally manufactured by local facilities who generate antivenoms specific for the viper species populating that area. Currently, no antivenoms have been officially licensed by the European Medicines Agency and no standardized protocols for clinical intervention are available^3^. Moreover, very little is known about the relative effectiveness of each antivenom against the viper’s venoms of a specific area, which often display highly heterogeneous compositions and cause variable clinical symptoms^5–7^. This limitation is particularly relevant considering the neurotoxic effects exerted by some species. Even though it occurs rarely, neurotoxicity has historically been associated with *V*. *ammodytes*^4,5^, as its venom contains “snake presynaptic PLA_2_ (phospholipase A2) neurotoxins” (SPANs), like vipoxin, vaspin and ammodytoxin. More recently, genetic and proteomic analyses have shown that also *V*. *aspis* venom contains PLA_2_ components which may be responsible for the neurological symptoms developed by bitten patients^5,6,8^. Interestingly, although PLA_2_s have been detected also in *V*. *berus* venoms^7,9,10^, there is general consensus in considering this adder as not neurotoxic^11^. This suggests that the PLA_2_s within *V*. *ammodytes*, *V*. *aspis* and *V*. *berus* venoms are functionally and possibly antigenically different and therefore may be variably susceptible to neutralization by available antisera.

In the present work, we performed a toxicological study on mice to compare the *in vivo* effects of *V*. *aspis* and *V*. *berus* venoms and to test the effectiveness of antivenoms commonly used in European hospitals (including in Italy) in counteracting their toxicity.

We found that the two venoms display very different compositions and cause remarkably different effects when locally injected in mice. *V*. *aspis* venom was indeed observed to exert a neuroparalytic action by causing a reversible degeneration of peripheral motor nerve terminals, whereas *V*. *berus* mainly leads to haemostatic imbalance. In addition, we found that the two tested antivenoms, despite efficiently contrasting the effect of *V*. *berus* venom, only barely neutralize *V*. *aspis* neurotoxicity. Our results suggest that differences in venom composition among viper species strongly influence the neutralization capability of antivenoms. The clinical implications of these observations are also discussed.

## Results

### *V*. *aspis* and *V*. *berus* venoms contain enzymatically active A2 phospholipases but only that one of *V*. *aspis* is neurotoxic

**Figure 1:**
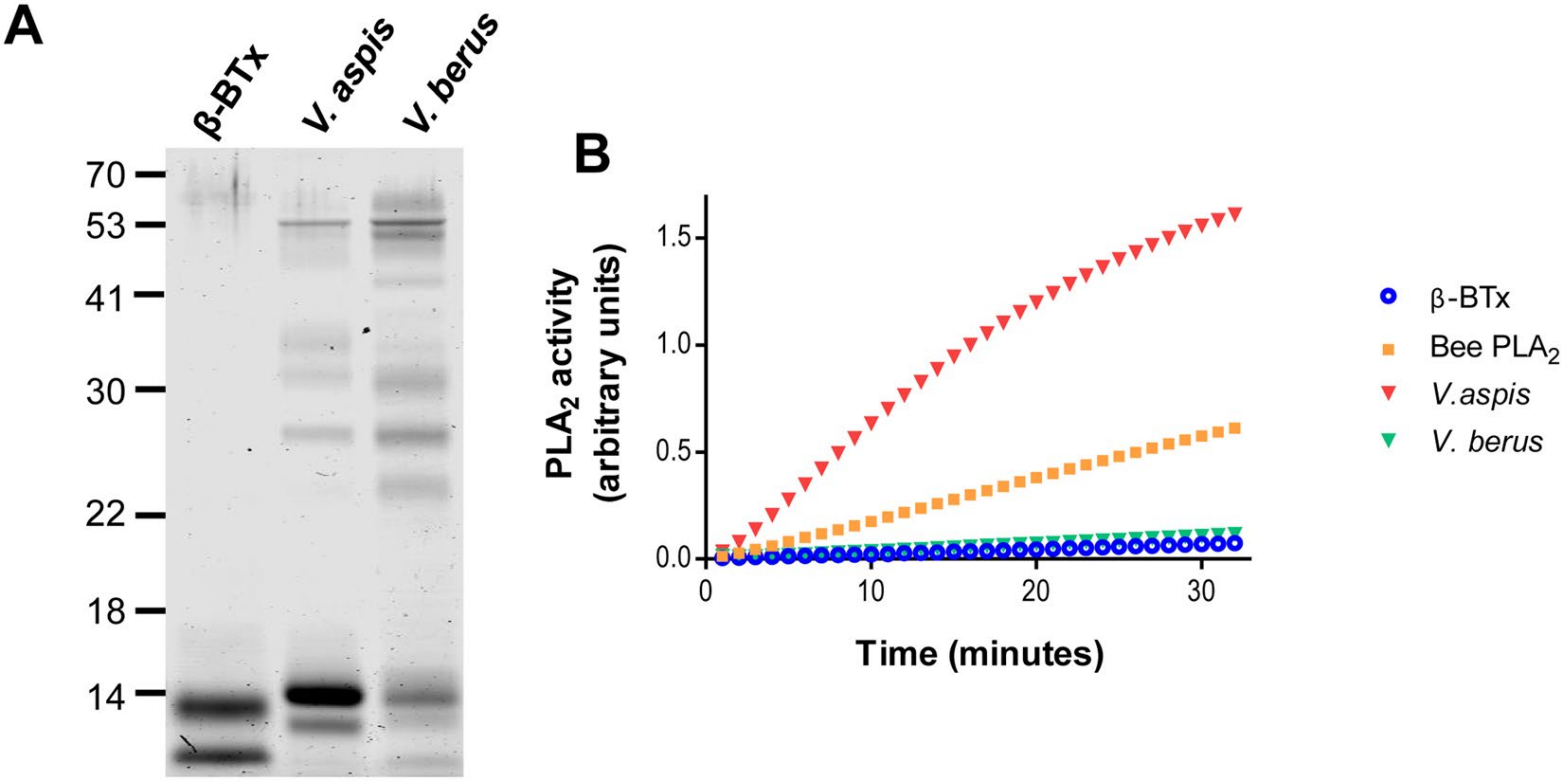
*V. aspis* and *V. berus* venoms display phospholipase A2 activity *in vitro*. (**A**) SDS-Page analysis showing the composition of *V*. *aspis* and *V*. *berus* venoms (1 µg per lane). Both venoms display a band around 14 kDa, typical of the phospholipase component. β-BTx, a neurotoxic phospholipase purified from *Bungarus multicinctus* (1 µg) is used as a reference. (**B**) PLA_2_ activities of *V*. *aspis* (10 ng, red triangles) and *V. berus* (10 ng, green triangles) venoms with respect to purified β-BTx (10 ng, blue circles). The activity of a purified PLA_2_ from Bee (10 ng, orange squares) is also shown as internal control of the assay. Traces are representative of a typical experiment done three times.

Figure 1A shows that the crude venoms of *V*. *aspis* and *V*. *berus* display different electrophoretic profiles, characterized by many components of different molecular weights. Nonetheless, in agreement with previous genetic and proteomic analyses, both venoms have a band around 14 kDa, typical of A_2_ phospholipases. As a reference, we loaded β-bungarotoxin (β-BTx) from the venom of *Bungarus multicinctus*, a very well characterized PLA_2_ toxin. To test whether these PLA_2_ are enzymatically active, we took advantage of an *in vitro* assay (Fig. 1B). We found that 10 nanograms of *V*. *aspis* venom display a remarkable phospholipase activity, even higher than that of β-BTx. At the same time, *V*. *berus* venom PLA_2_ activity is significantly lower, even though still comparable to β-BTx.

**Figure 2:**
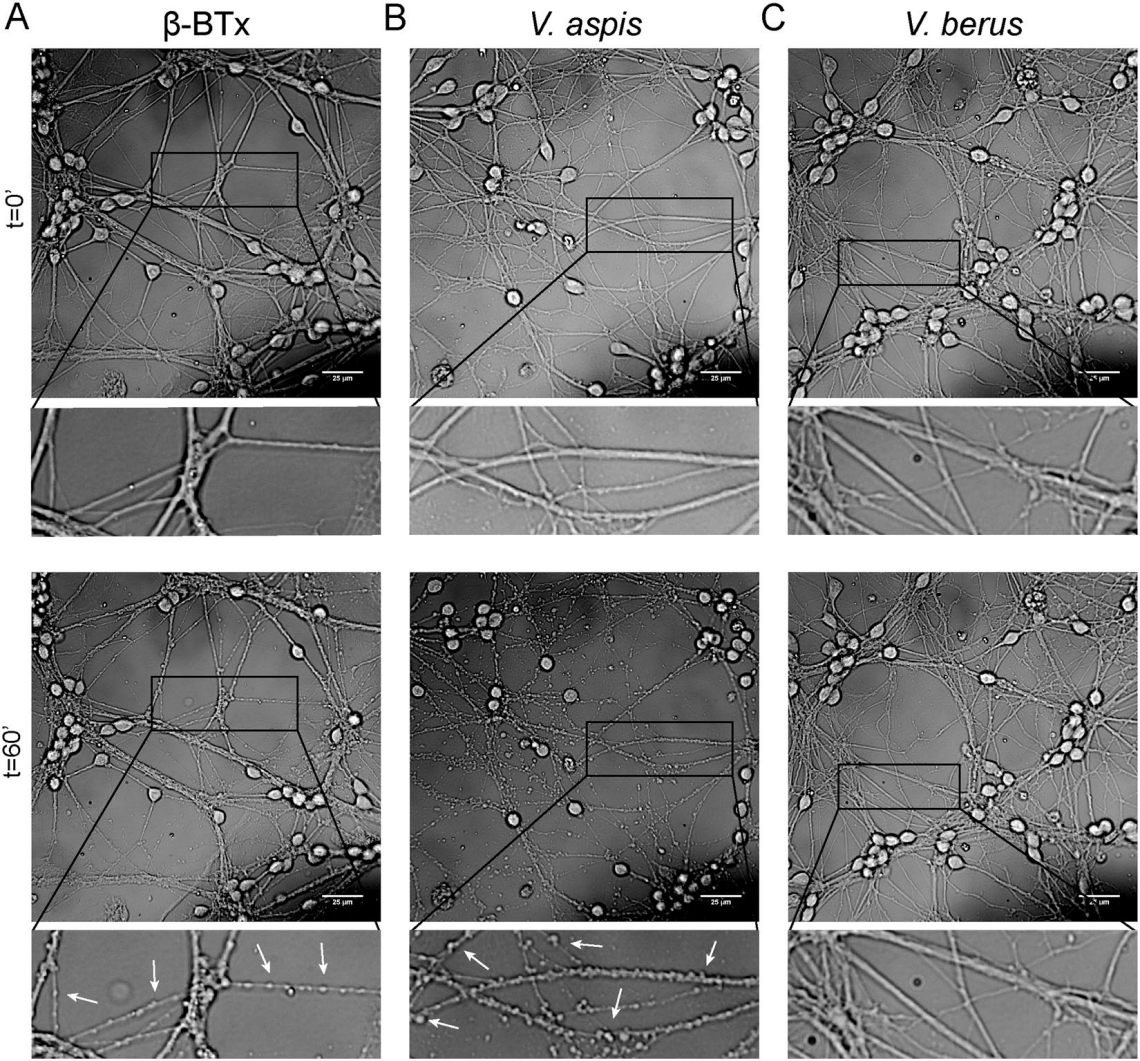
Live imaging of CGNs asses the presence of neurotoxic PLA2 in *V. aspis*, but not in *V. berus*, venom. CGNs were treated with (**A**) β-BTx (0.25 µg/ml, 12,5 nM) or (**B**) V. *aspis* venom (35 µg/ml) or (**C**) *V*. *berus* venom (35 µg/ml) and pictures taken at indicated time points. *V*. *aspis*, but not *V*. *berus*, venom induces a bulging effect similar to β-BTx, hallmark of neurotoxic PLA_2_. Pictures show most significant frames coming from Supplementary Movie M1, M2 and M3 and are representative of a typical time course. Scale bar = 30 μm.

Since the neurological symptoms caused by *V*. *aspis* envenomation are believed to be due to the presence of a neurotoxic PLA_2_, we investigated whether this venom induces the typical “bulging effect” exerted by SPANs on cultured neurons. This phenomenon consists in the formation of discrete varicosities at various sites along neuronal axons and projections as a consequence of increased vesicles exocytosis and altered plasma membrane permeability^12–14^. Figure 2A and Supplementary Movie M1 show this peculiar pathological phenotype in cerebellar granule neurons (CGNs) incubated with 10 nM β-BTx. *V*. *aspis* venom (35 µg/ml) causes neurites bulging in a time course (60 minutes) and with a frequency similar to β-BTx (Fig. 2B and Supplementary Movie M2). Importantly, the same effect is recapitulated by the fraction of *V*. *aspis* venom containing the PLA_2_ component (Fig. S1 and Supplementary Movie M4), isolated by size exclusion chromatography, but not by other fractions reunited after chromatography (Fig. S1B and supplementary Movie M5), supporting the idea that bulges formation is a direct consequence of the phospholipase activity. On the other hand, Fig. 2C shows that the venom of *V*. *berus* (35 µg/ml) induces very little, if any, bulging on CGNs, even when the incubation time is prolonged to 90 minutes (Supplementary Movie M3).

All together, these observations indicate that both *V*. *aspis* and *V*. *berus* venoms contain enzymatically active PLA_2_ but that only the former induces the neurotoxic phenotypical effect peculiar of SPANs on cultured neurons.

### *V*. *aspis* venom causes neuromuscular paralysis via degeneration of motor nerve terminals

**Figure 3:**
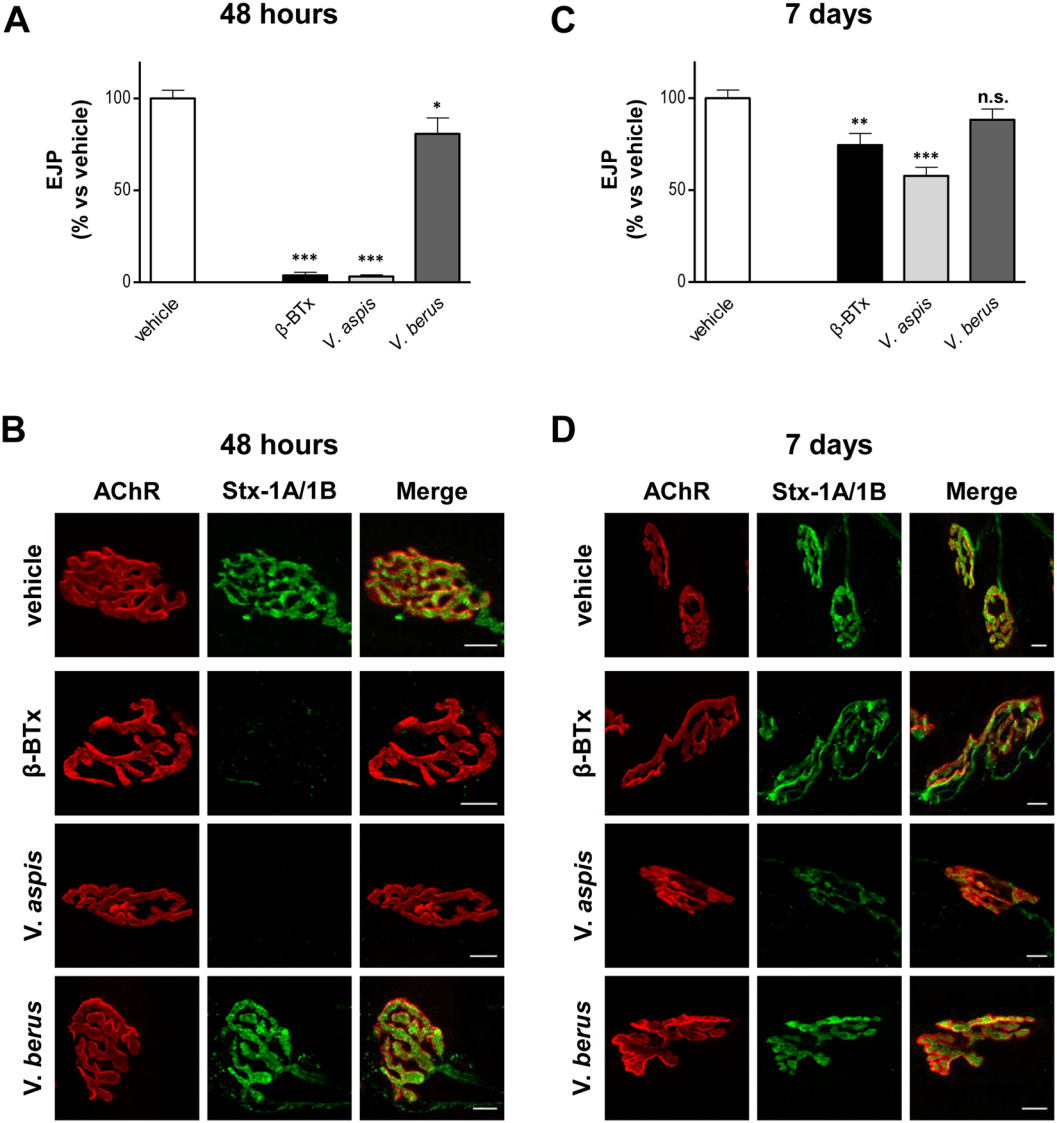
*V*. *aspis* venom, but not *V*. *berus* venom, causes reversible neuromuscular paralysis due to NMJ degeneration. Electrophysiological recordings of EJP from mouse solei (**A**) 48 h and (**C**) 7 days after injection of β-BTx (10 µg/Kg) or of indicated viper venom (100 µg/Kg). Bars represent the average EJP amplitude of 15 fibres per muscle from at least three different mice per condition, expressed as a percentage of control condition (injection of the vehicle alone); paired *t*‐test, ^*^p < 0.01, ^**^p < 0.001, ^***^p < 0.0001 *versus* control (vehicle); error bars represent s.e.m.; n.s. = not significant. After electrophysiology, soleus muscles were imaged for the presynaptic markers syntaxin-1A/1B (green) and for the postsynaptic ACh receptors (AChR, red) to evaluate NMJ integrity, (**B**) 48 h and (**D**) 7 days post injection. Scale bar = 10 μm.

Clinical data suggest that the main neurological symptom on humans upon *V*. *aspis* envenomation is a neuromuscular blockade^5^. An accurate read out to assay the neurotoxicity of SPANs is the electrophysiological measurement of Evoked Junction Potential (EJP) amplitudes on isolated soleus muscles, following *in vivo* injection of the venom in the mouse hind limb^15,16^. To this aim, we intramuscularly administered 100 µg/kg of *V*. *aspis* venom, a sub-lethal amount roughly corresponding to 1/7 of the LD_50_ (according to^17^). In parallel, a group of mice was injected with β-BTx (5 µg/kg) at the same limb site and served as positive control for neurotoxicity. We evaluated the soleus EJPs 48 hours after the injections (acute phase) and found that *V*. *aspis* venom causes a complete blockade of nerve-muscle transmission, in a way very similar to β-BTx (Fig. 3A). *V*. *berus* venom (100 µg/kg, roughly 1/5 of the LD_50_) was also tested for peripheral neurotoxicity, but as shown in Fig. 3A it does not significantly alter the capability of motor nerve endings to elicit EJPs in muscle fibres. This evidence strongly suggests that *V*. *berus* venom is not neurotoxic.

The presence of PLA_2_ components in its venom, encourages the idea that *V*. *aspis* neurotoxicity may be due to a SPAN-like toxin causing the degeneration of motor nerve terminals^16,18–20^, yet this possibility has never been investigated in detail. Degeneration of motor nerves can be easily monitored via neuromuscular junction (NMJ) immunohistochemistry^14,15,21,22^. Accordingly, soleus muscles processed for electrophysiology were subsequently stained for the presynaptic marker syntaxin-1A/1B (Stx-1A/1B), in order to assess the integrity of poisoned nerve terminals. We also took advantage of a fluorescent α-BTx, which specifically binds nicotinic acetylcholine receptors (AChR), to spot NMJs and to evaluate the status of post-synaptic motor end-plates. Figure 3B shows that in the acute phase of intoxication, *V*. *aspis* venom, like β-BTx, causes the complete degeneration of nerve terminals as evidenced by the disappearance of Stx-1A/1B staining, otherwise very intense and sharp. At the same time, the post synaptic compartment appears unaffected, as AChRs remain densely clustered and maintain the typical pretzel-like shape. Consistent with functional neurotransmission (Fig. 3A), NMJs of soleus muscles treated with *V*. *berus* venom display intact nerve terminals and no alteration of the post synaptic compartment. Nevertheless, in *V*. *berus*-treated animals we noticed a remarkable anticoagulant effect spreading all along the injected lower hind limb, a phenomenon not observed in *V*. *aspis*-injected mice (Fig. S2).

Like in the case of β-BTx (and other SPANs), muscles intoxicated by *V*. *aspis* venom restore nerve-muscle transmission with time (Fig. 3C). Functional recovery is achieved in one week thanks to the remarkable plasticity of vertebrate NMJ, which can reform even after complete degeneration. As shown in Fig. 3D, 7 days after intoxication with β-BTx and *V*. *aspis*, nerve terminals have regrown and re-innervated the muscle fibres, forming new NMJs looking and performing like the original ones.

Altogether, these results demonstrate that *V*. *aspis*, but not *V*. *berus* venom, exerts a neurotoxic action by causing the degeneration of peripheral nerve terminals at the NMJ. However, as already reported for other SPANs, this effect is completely reversible, and a fully functional neuromuscular synapse re-forms within few days.

### Antisera barely protect from *V*. *aspis* neurotoxicity but neutralize the anticoagulant effect of *V*. *berus*

**Figure 4:**
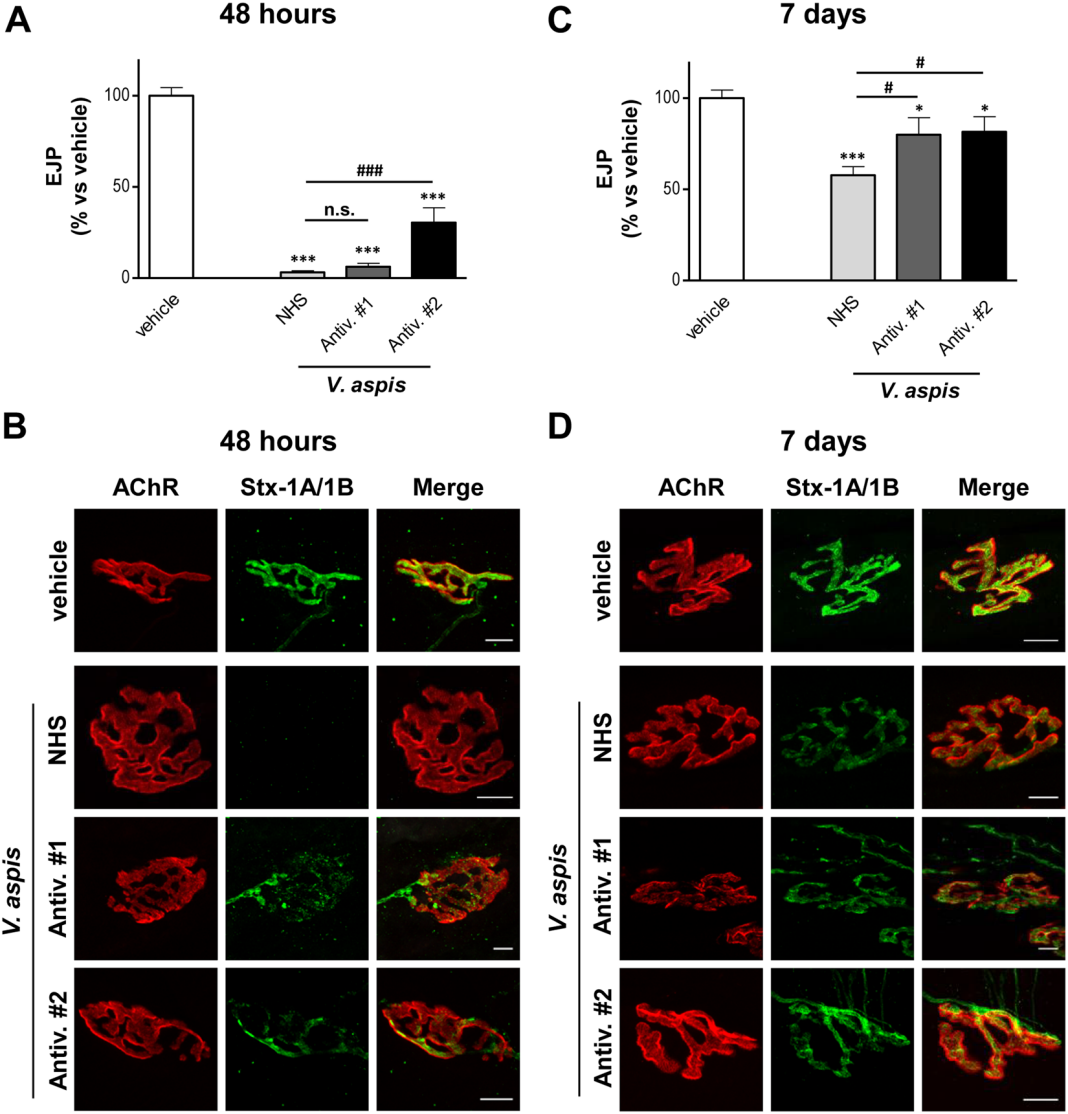
Acute neurotoxicity of *V. aspis* is barely prevented by antivenoms. Electrophysiological recordings of EJP from mice solei (**A**) 48 h or (**B**) 7 days after the injection of *V*. *aspis* venom (100 µg/Kg) pre-incubated with Antivenom#1 or Antivenom#2 or NHS (Normal Horse Serum) (see methods). Bars represent the average EJP amplitude of 15 fibres per muscle from at least three different mice per condition, expressed as a percentage of control condition (injection of the sole vehicle); paired *t*‐test, ^*^p < 0.01, ^**^p < 0.001, ^***^p < 0.0001 *versus* control (vehicle) or ^#^*p* < 0.01, ^##^*p* < 0.001, ^###^*p* < 0.0001 *versus* NHS; error bars represent s.e.m.; n.s. = not significant. After electrophysiology, soleus muscles were imaged for the presynaptic markers syntaxin-1A/1B (green) and for the postsynaptic ACh receptors (AChR, red) to evaluate NMJ integrity (**B**) 48 h or (**D**) 7 days. Scale bar = 10 μm.

Once characterized the mechanism responsible for *V*. *aspis* neurotoxicity, we investigated whether antivenoms commonly administered in hospitals can counteract this activity. We used two equine antisera raised against either *V*. *ammodytes* (Antivenom#1) or *V*. *berus* (Antivenom#2)^3^. To test their activity in the most favourable condition, we pre-incubated the venoms with antisera and then injected the mixtures *in vivo*, using electrophysiology and NMJ imaging to evaluate the neutralizing effect. We used a 3:1 antivenom/venom-LD_50_ ratio for both antisera and normal horse serum as a control. In the acute phase of intoxication, Antivenom #1 neither prevented neurotransmission impairment (Fig. 4A) nor protected nerve terminals from degeneration (Fig. 4B). No substantial improvements were observed even when the antivenom/venom-LD_50_ ratio was increased up to 15:1. (Fig. S3A and B). On the other hand, nerve endings of muscles intoxicated with *V*. *aspis* pre-incubated with Antivenom #2 were able to elicit EJPs in response to nerve stimulation 48 hours after injection (Fig. 4A). However, average EJP amplitude was largely lower than control muscles (injected with vehicle), indicating that the neutralization effect of this second antivenom, even though higher than Antivenom #1, is partial. Consistently, Stx-1A/1B staining is still detectable at NMJs but appears very faint and irregular (Fig. 4B).

Despite barely effective in the acute phase, both antisera seem to slightly speed up the functional recovery in a later stage of intoxication. Muscles injected with venom-antisera mixtures display higher EJPs than muscles injected with venom-NHS mixture  after one week (Fig. 4C), and nerve terminals exhibit a brighter Stx-1A/1B signal (Fig. 4D). This is more evident for Antivenom #1 when used in the higher amount (Fig. S3C and D).

These results suggest that the two antisera have limited capability to prevent the acute neurotoxic effects of *V*. *aspis* venom, even when used in the most favourable conditions for neutralization (i.e. pre-incubation with venoms) and at high concentration. However, they appear to slightly accelerate the time course of recovery.

Interestingly, both antisera can successfully prevent the haemorrhagic effect caused by *V*. *berus* venom (Fig. S4). Despite the existence of different anticoagulant mechanisms, the most likely explanation is the inhibition of coagulation factors by a non-catalytic action of the PLA_2_^9,23,24^. Our results suggest that the tested antisera neutralize at different extent the PLA_2_ of the two venoms, being very efficacious against the anticoagulant activity of *V*. *berus* yet really ineffective against the neurotoxic activity of *V*. *aspis*^25,26^. To test whether this derives from a different immunoreactivity of the antisera against the PLA_2_ of the two venoms, we performed a western blot analysis using antivenoms as primary antibodies. Consistently with our *in vivo* experiments, we found that both antisera react more avidly with *V*. *berus* PLA_2_ component than that of *V*. *aspis* (Fig. S5). Moreover, the PLA_2_ of *V*. *aspis* venom is bound more efficiently by Antivenom #2 than Antivenom #1, a result in keeping with its slightly higher ability to block *V*. *aspis* neurotoxicity.

## Discussion

Effectiveness of antivenom administration in counteracting viper envenoming is poorly clear^3^. One of the reasons is the existence of many different viper subspecies, whose venoms are extremely variable in term of biochemical composition and pathogenic mechanism of action^5–7^. Moreover, the available antisera are generally raised against one specific venom, which may limit cross neutralization.

In the present paper, we compared the *in vivo* activity of *V*. *aspis* and *V*. *berus* venoms harvested in north Italy, one of the few areas where different species of viper snakes coexist. Furthermore, the ability of antivenoms commonly available in European hospitals to counteract the specific effects of these venoms was evaluated^3,4^. To the best of our knowledge this is the first time that such an analysis is performed *in vivo*. A main difference we found is that *V*. *aspis*, but not *V*. *berus* venom, is neurotoxic and causes neuromuscular paralysis. Despite this was already known from previous studies and clinical data^5,6,27^, we show here for the first time that this is due the selective degeneration of peripheral motor nerve terminals. This activity is ascribable to the PLA_2_-containing fraction of the venom and is very similar to the pre-synaptic neurotoxicity of β-BTx: accordingly, the PLA_2_ of *V*. *aspis* has to be considered a SPAN^19,28^. As demonstrated by functional and morphological assays, NMJ degeneration is completely reversible since nerve terminals rapidly regenerate to look and perform like the original ones. This evidence agrees with clinical data on humans showing a complete recovery of neurologic symptoms after neurotoxic viper envenomation^5^.

Conversely, the venom of *V*. *berus* does not induce any neuromuscular impairment, at least in our experimental model. The main effect caused by *V*. *berus* injection was an evident and widespread coagulopathy, not shared by *V*. *aspis* venom, consistent with the presence of anticoagulant and haemorrhagic toxins reported in previous studies^7,29^. This effect is in part due to the PLA_2_ component but in a way not necessarily dependent on its enzymatic activity^9,23,24^. Our results are in line with the common belief that *V*. *berus* venom is devoid of neurotoxic activity, yet some *V*. *berus* subspecies of eastern Europe have been shown to cause neurological effects on animal models and humans^10,30^. This further suggests that not only heterotypic but also intratypic variations in the composition of viper venoms can occur.

The efficacy of an antivenom depends on diverse factors, including the inter- and intra-species variability of snake venom composition. Our experiments clearly show that the two tested antivenoms efficiently counteract the anticoagulant activity of *V*. *berus* but barely prevent the acute neurotoxic effect of *V*. *aspis* venom, only accounting for a moderately faster recovery of neurotransmission in a later stage of intoxication. This might be due to a low affinity of the antibodies contained in the antisera, raised against either *V*. *ammodytes* (Antivenom #1) or *V*. *berus* (Antivenom #2), for the PLA_2_ of *V*. *aspis*, not sufficient to prevent completely neurotoxin activity on the presynaptic plasma membrane, but useful to facilitate the elimination of the toxin from the body, thus decreasing toxin concentration at NMJ. In this way, nerve endings may be attacked by less SPANs and undergo a partial degeneration, enough severe to cause block of neurotransmission, but also fixable much faster with respect to a complete degeneration.

In conclusion, our results strongly suggest that antisera display variable and possibly insufficient neutralization capability against the different toxic activities present in the venoms of different viper subspecies. Our findings are supported by “venomics” studies showing that, despite being closely related phylogenetically, snake venom compositions and protein sequences of homologous toxins are generally characterized by remarkable diversity^7,31^. Although in our study, due to a supply shortage, we could not test the unique marketed antivenom raised against all the three main viper species present in Europe (*V*. *aspis*, *V*. *berus and V*. *ammodytes*)^3^, we provide here an explanation for the lack of clarity about the real effectiveness of viper antivenoms, reinforcing the idea of using polyvalent antivenoms. In this scenario, a pan-viper antivenom raised against the many different viper species and subspecies may represent an attractive strategy for a more effective handling of European snakebite patients. Furthermore, small molecule inhibitors targeting the activity of key toxic components of venoms, notably PLA_2_s or matrix metalloproteases, may also be considered for the development of novel wide-spectrum antivenoms^32,33^. This is particularly relevant because antivenom choice in hospitals is generally made according to the momentary availability rather than considering the viper species responsible for biting, that is, in any case, difficult (if not impossible) to be ascertained by clinicians in the emergency room.

## Materials and Methods

Venoms were milked from *V*. *aspis aspis* or *V*. *berus berus* snakes harvested in Northern Italy. Antivenom#1 and Antivenom#2 were provided by the Poison Control Centre and National Toxicology Information Centre of Pavia, ICS Hospital. SimplyBlue^TM^ SafeStain is from Invitrogen. Cytosine β-D-arabinofuranoside hydrochloride (C6645), DNAse I from bovine pancreas (DN25), poly-L-lysine hydrobromide (P1274), Trypsin (T4799) and β-BTx are from Sigma Aldrich. µ-Conotoxin GIIIB is from Alomone, Jerusalem, Israel. Protease inhibitors cocktail is from Roche. NuPage 12% Bis-Tris gels and MES buffer are from Life technologies. Protran nitrocellulose membranes is from Whatman. Luminata^TM^ is from Merck Millipore. Primary antibodies: anti syntaxin-1A1B polyclonal antibodies have been produced in our laboratory. Secondary antibodies for western blotting are from Calbiochem^®^; secondary antibodies for immunofluorescence and α-bungarotoxin-Alexa 555 are from Thermo Scientific, Waltham, MA, USA.

### SDS-Page and Western blotting

Protein amount in whole venoms was determined via BCA Protein Assay kit (Pierce^TM^, Thermo Scientific). 1 µg of β-BTx or of *V*. *aspis* or of *V*. *berus* venom was diluted in Laemmli sample buffer and heat-denaturated. Samples were loaded into NuPage 4–12% Bis-Tris gels and separated by electrophoresis in MES buffer. Proteins were visualized using SimplyBlue^TM^ SafeStain. For Western blotting, proteins were transferred onto Protran nitrocellulose membranes and saturated for 1 h in PBS-T (PBS, 0.1% Tween 20) supplemented with 5% non-fatty milk. Incubation with Antivenom#1 or Antivenom#2 was performed overnight at 4 °C. Thereafter, membranes were washed three times with PBS-T and incubated with anti-Horse HRP-conjugated secondary antibodies for 1 h. Membranes were washed three times with PBS and revealed with Luminata^TM^ using an Uvitec gel doc system (Uvitec Cambridge).

### *In vitro* phospholipase Activity

The phospholipase activity of whole venoms (10 ng) and of β-BTx (10 ng) was assayed as previously described^12^ with a cPLA_2_ assay kit (Cayman Chemical) and according to manufacturer’s instruction.

### Primary cultures of Cerebellar Granule Neurons and intoxication assay

Primary cultures of rat cerebellar granule neurons (CGNs) were prepared from 6- to 8-days-old rats as previously described^34^. At 6–8 days *in vitro* complete culture medium was changed with KRH (NaCl 125 mM; KCl 5 mM; HEPES 25 mM; CaCl_2_ 2 mM MgSO_4_ 1.2; KH_2_PO_4_ 1.2 mM; glucose 6 mM) supplemented with β-BTx (0.25 µg/ml), or *V*. *aspis* venom (35 µg/ml) or *V*. *berus* venom (35 µg/ml). As previously described^12^, neurotoxicity was evaluated by following the formation of bulges in live imaging using DMI6000 inverted epifluorescence microscope (Leica) equipped with a 63x HCX PL APO oil immersion objective NA 1.4. Images were acquired with an Orca-Flash4 digital camera (Hamamatsu).

In a series of experiments, *V*. *Aspis* venom was fractionated by size exclusion chromatography using a Superdex 200 10/300 column (GE Healthcare) in buffer 15 mM TRIS-HEPES, 125 mM NaCl, pH 7.3. Five fractions corresponding to the main peaks were collected and analyzed by SDS-PAGE. The peak containing the band corresponding to the putative phospholipase was quantified and added to cultured neurons as indicated in Fig. S1. As a control, the other peaks were re-mixed, quantified and added to neurons (reported in the text as “other fractions”).

### Electrophysiological recordings of evoked junction potential

Electrophysiological recordings were performed as described in^35^. Swiss-Webster adult female CD1 mice weighing 20–25 grams were injected in the left hind limb with β-BTx (10 µg/Kg), *V*. *aspis* venom or *V*. *berus* venom (100 µg/Kg), alone or mixed with Antivenom#1 or Antivenom#2 or normal horse serum (NHS), diluted in vehicle (0.9% NaCl 0.2% gelatine). The amount of Antivenom#1 was set in order to achieve 3X or 15X excess of neutralization capacity, calculated considering the specific neutralization capacity towards *V*. *aspis* or *V*. *berus* reported by the manufacturer. For Antivenom#2 the specific neutralization capacity was not made available by the manufacturer; therefore venoms were directly diluted in the serum. At scheduled times, mice were euthanized and soleus muscles dissected. Electrophysiological recordings were performed in modified Ringer’s solution (NaHCO_3_ 12 mM; KCl 4 mM; KH_2_PO_4_ 1 mM; NaCl 138.8 mM; MgCl_2_ 1 mM; CaCl_2_ 2 mM, buffered to pH 7.4 by bubbling a gas mixture 95% O_2_, 5% CO_2_) using intracellular glass microelectrodes (WPI) filled with 1 M KCl and 2 M CH_3_COOK. Evoked junction potentials (EJP) were recorded in current-clamp mode, starting from resting membrane potential of −70 mV, adjusted with direct current injection (if needed). EJP were elicited by supramaximal nerve stimulation at 0.5 Hz, using a suction microelectrode connected to a S88 stimulator (Grass, Warwick, RI, USA). Muscle contraction was prevented by 1 µM µ-Conotoxin GIIIB. Signals were amplified with intracellular bridge mode amplifier (BA-01X; NPI, Tamm, Germany), sampled using a digital interface (NI PCI-6221; National Instruments, Austin, TX, USA) and recorded by means of electrophysiological software (WinEDR; Strathclyde University, Glasgow, Scotland, UK). EJP measurements were carried out with Clampfit software (Molecular Devices, Sunnyvale, CA, USA). EJP represent the average value obtained analysing at least three muscles (15 fibers/muscle) for each condition at each time-point and reported as a percentage with respect to control muscles.

### Imaging of neuromuscular junctions

After electrophysiological recordings, muscles were fixed in 4% paraformaldehyde in PBS for 10 min at RT. Each soleus muscle was separated in bundles of about 20–40 fibres to facilitate the staining. Samples were quenched in 50 mM NH_4_Cl in PBS and treated for 2 h with blocking solution (15% vol/vol goat serum, 2% wt/vol BSA, 0.25% wt/vol gelatine, 0.2% wt/vol glycine 0.5% Triton X-100 in PBS). Thereafter, incubation with anti syntaxin-1A/1B (1:200) primary antibody was carried out as previously described^36^. Muscles were then extensively washed and incubated with a secondary antibody conjugated with Alexa-488 diluted in blocking solution (1:200) supplemented with α-bungarotoxin-Alexa 555 (1:200) to counterstain post-synaptic nicotinic acetylcholine receptors (AChR). Images were collected with a Leica SP5 confocal microscope (Leica Microsystems, Wetzlar, Germany) equipped with 100X HCX PL APO NA 1.4 objective. Laser excitation line, power intensity, and emission range were chosen according to each fluorophore in different samples to minimize bleed-through.

### Ethics statements

All experiments were performed in accordance with the Italian laws and policies (D.L. n°26 14th March 2014) and with the guidelines established by the European Community Council Directive n° 2010/63/UE and approved by the veterinary services of the University of Padova (O.P.B.A.—Organismo Preposto al Benessere degli Animali) (protocol 359/2015).

## Electronic supplementary material

Supplementary Figures file

Supplementary movie M1 - β-BTx

Supplementary movie M2 - V. aspis

Supplementary movie M3 - V.berus

Supplementary movie M4 - V. aspis PLA2 fraction

Supplementary movie M5 - V. aspis other fractions
