# Supplementary material for: Variability in venom composition of European viper subspecies limits the cross-effectiveness of antivenoms
Source: Sci Rep. 2018 Jun 29;8:9818. doi: 10.1038/s41598-018-28135-0 (PMC6026201; doi:10.1038/s41598-018-28135-0)
Supplement: Supplementary file 1 — Supplementary Figures file [file 41598_2018_28135_MOESM1_ESM.pdf]

Variability in venom composition of European viper subspecies limits the cross-effectiveness of antivenoms.

Giulia Zanetti, Elisa Duregotti, Carlo Alessandro Locatelli, Andrea Giampreti, Davide Lonati, Ornella Rossetto and Marco Pirazzini

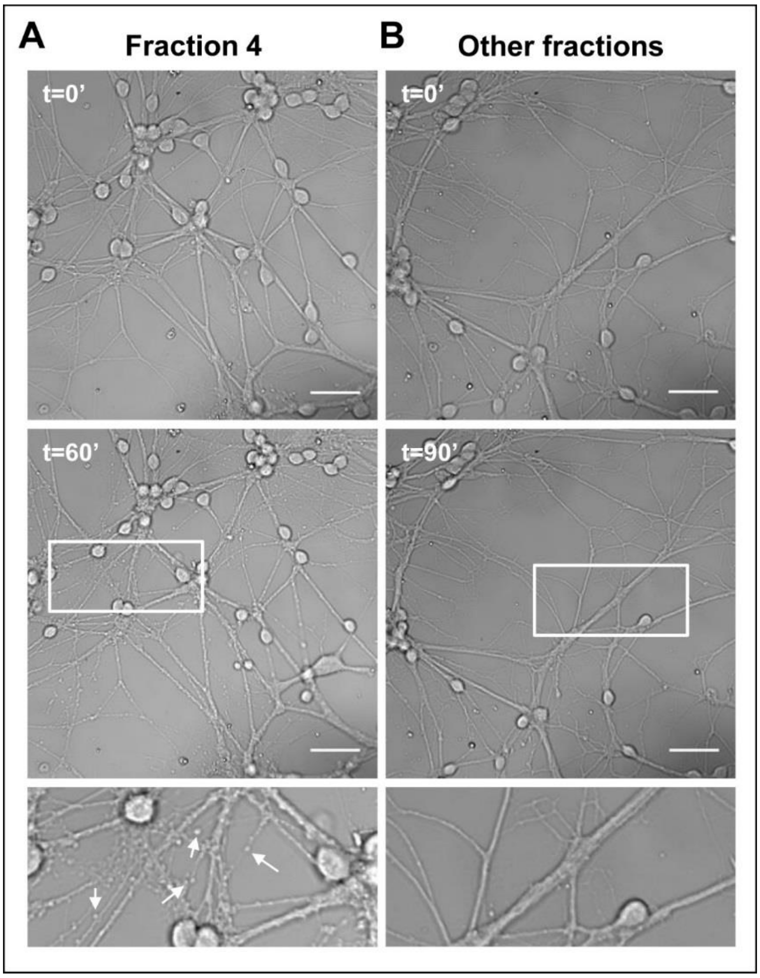

Fig S1. ***V. aspis*** **neurotoxicity is retained by the venom fraction containing the PLA_2 component.** The venom of *V. Aspis* was fractionated by size exclusion chromatography and CGNs were treated with **(A)** the fraction containing the PLA_2 component (0.25 µg/ml) or **(B)** all other fractions re-mixed together (0.25 µg/ml). Only the PLA_2 fraction causes axon bulges. Pictures show most significant frames coming from supplementary Movie M4 and M5 and are representative of a typical time course. Scale bar=30 µm.

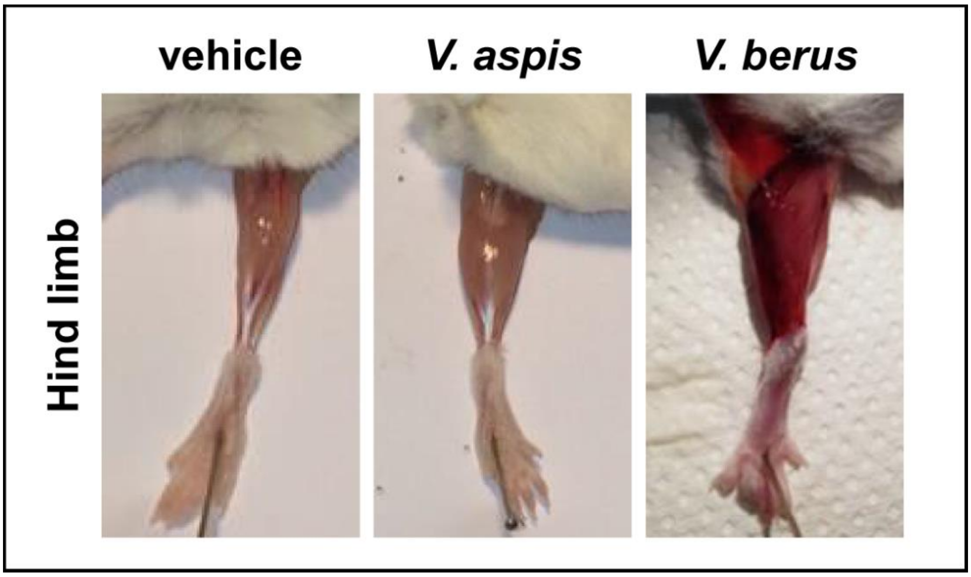

Fig S2. ***V. berus*** **causes haemostatic imbalance upon** ***in vivo*** **injection.** Vehicle or *V. aspis* venom (100 µg/Kg) or *V. berus* venom (100 µg/Kg) were injected at the level of the hind paw. After 48 hours the limb was skinned. *V. berus* venom causes a copious haemorrhage, whereas *V. aspis* does not induces any macroscopic effects.

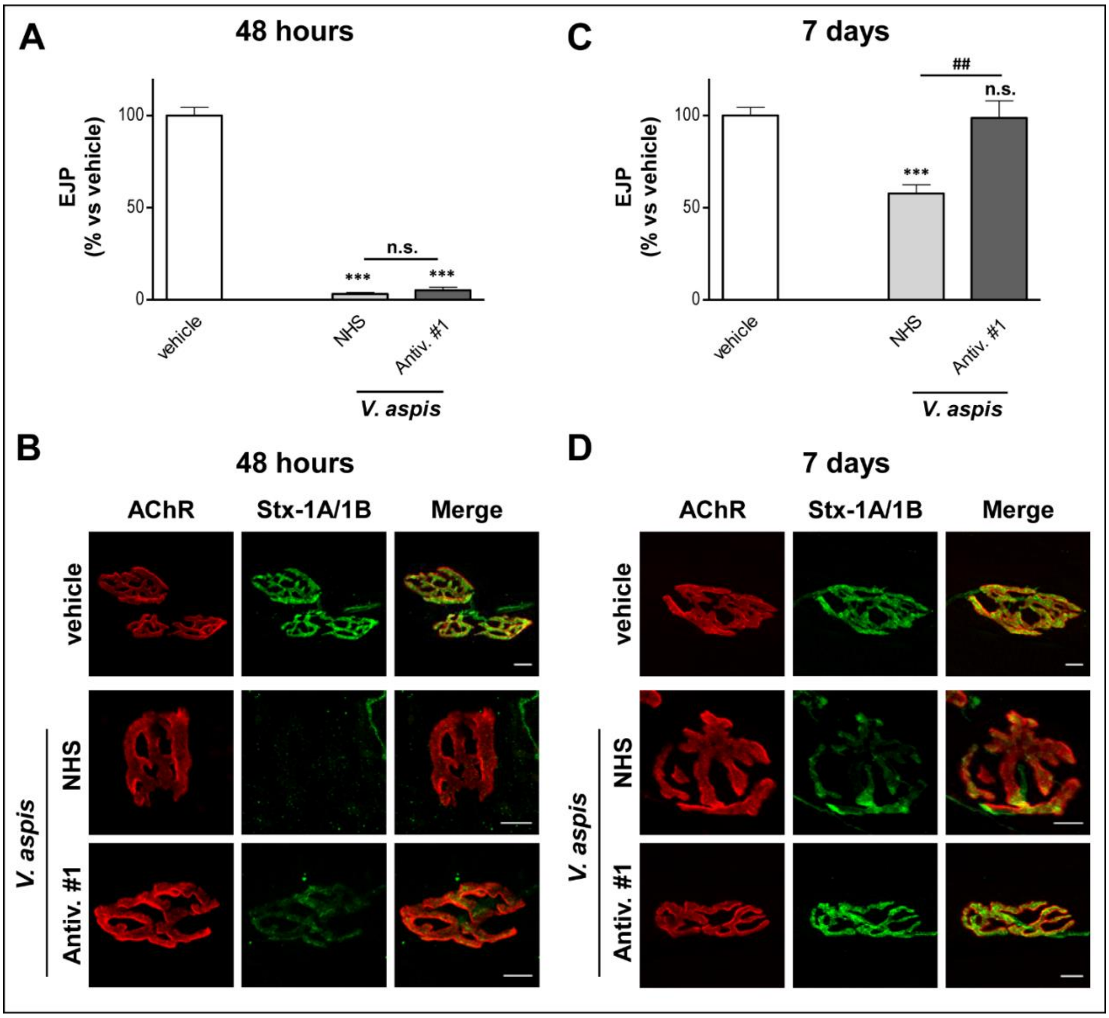

Fig S3. High amount of Antivenom#1 slightly speeds up recovery from neuromuscular paralysis. Electrophysiological recordings of EJP from mice solei **(A)** 48 h or **(B)** 7 days after injection in the hind limb of *V. aspis* venom (100 µg/Kg) pre-incubated with Antivenom #1 (15:1 antivenom/venom-LD_{50} ratio) or NHS. Bars represent the average EJP amplitude of 15 fibres per muscle from at least three different mice per condition, expressed as a percentage of control EJP (injection of the sole vehicle); paired *t*-test, *p<0.01, **p<0.001, ***p<0.0001 *versus* control (vehicle) or #p<0.01, ##p<0.001, ###p<0.0001 *versus* NHS; error bars represent s.e.m.; n.s.=not significant. After electrophysiology, soleus muscles were imaged for the presynaptic markers syntaxin-1A/1B (green) and for the postsynaptic ACh receptors (AChR, red) to evaluate NMJ integrity **(B)** 48 h or **(D)** 7 days post injection. Scale bar=10 µm.

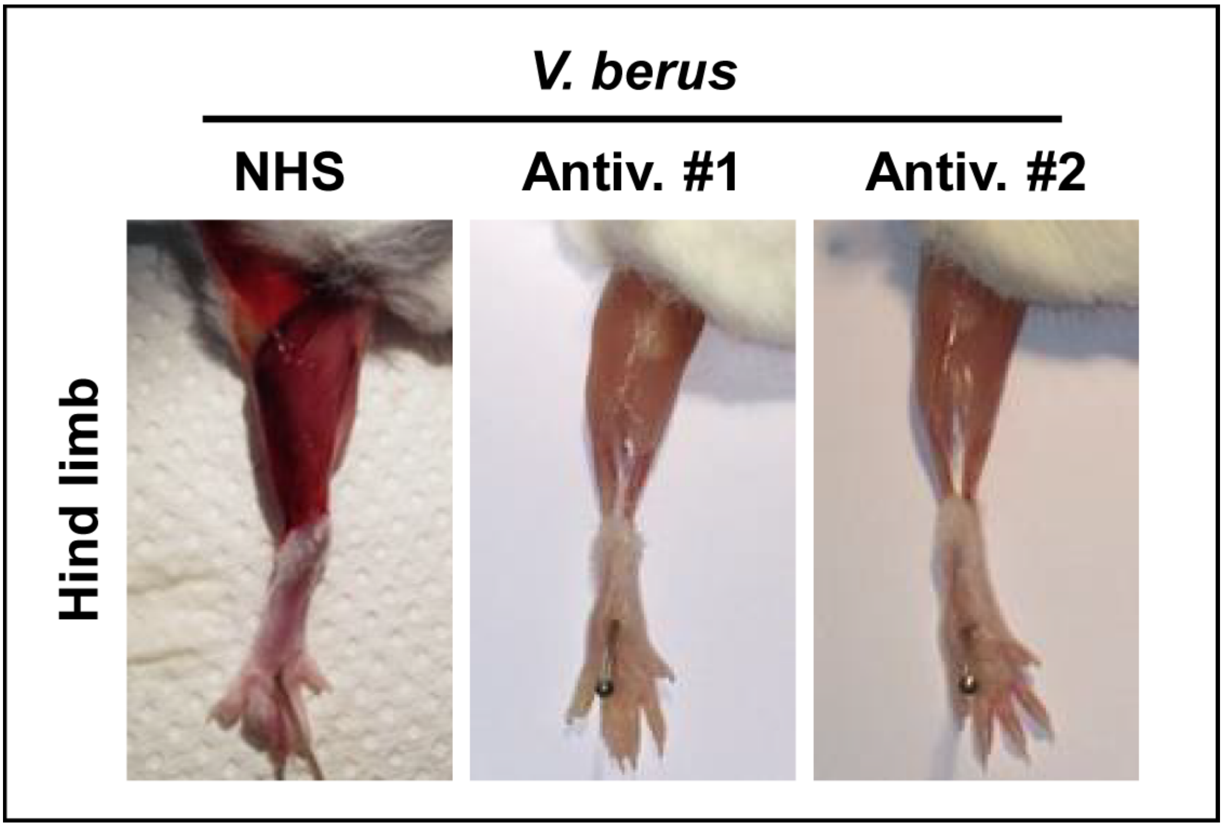

Fig S4. Antivenoms neutralize the haemorrhagic effect of *V. berus* venom. *V. berus* venom (100 µg/Kg) pre-incubated with NHS or Antivenom #1 or Antivenom #2 was injected at the level of the hind paw. After 48 hours the limb was skinned and the haemorrhagic effect evaluated.

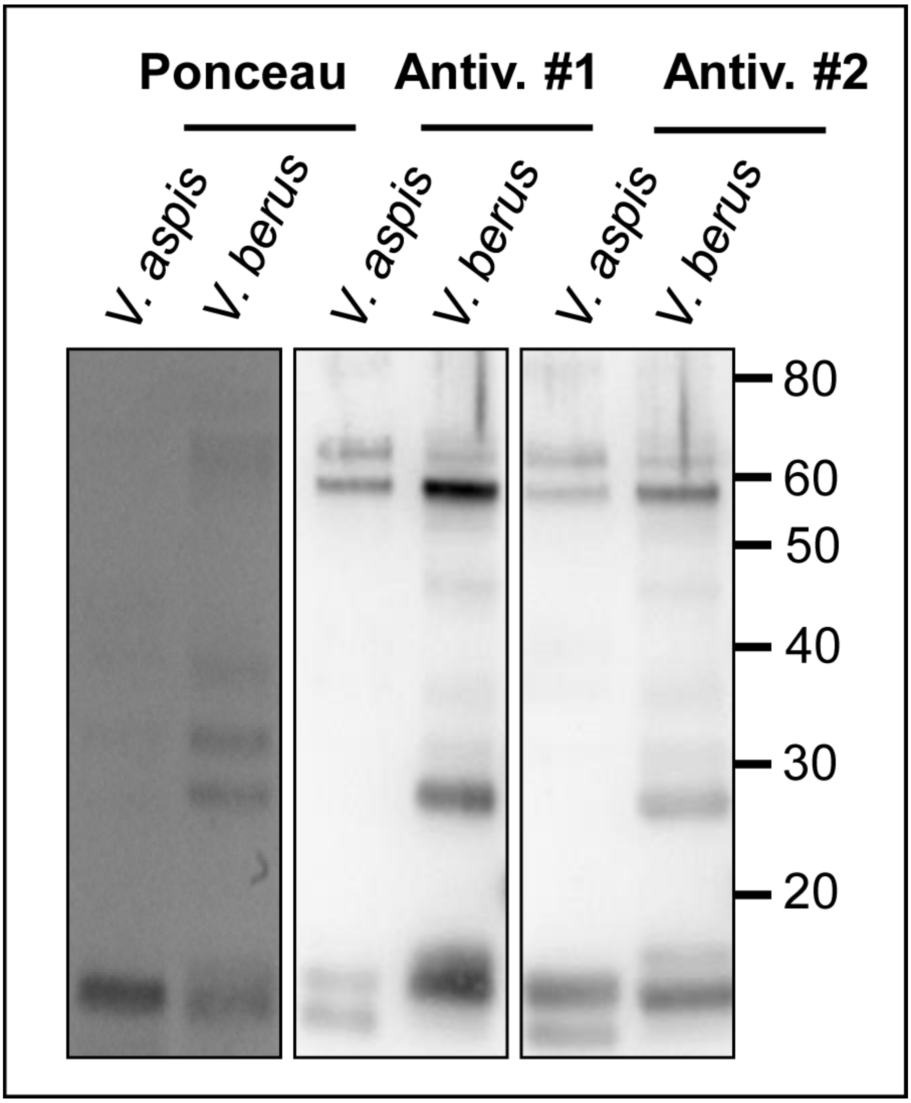

Fig S5. Western blotting analysis of whole venoms with antisera. 0.75 µg/well of *V. aspis* or of *V. berus* venom were separated in a reducing SDS-PAGE and then transferred on a nitrocellulose membrane for immunoblotting. Red Ponceau staining shows the composition of whole venoms. Antivenom #1 recognizes very poorly *V. aspis* venom, especially the PLA_2 component which is the most abundant component. Antivenom #2 displays a slightly higher affinity for the PLA_2 component of *V. aspis* venom. Both sera have higher affinity for *V. berus* than *V. aspis* venom.

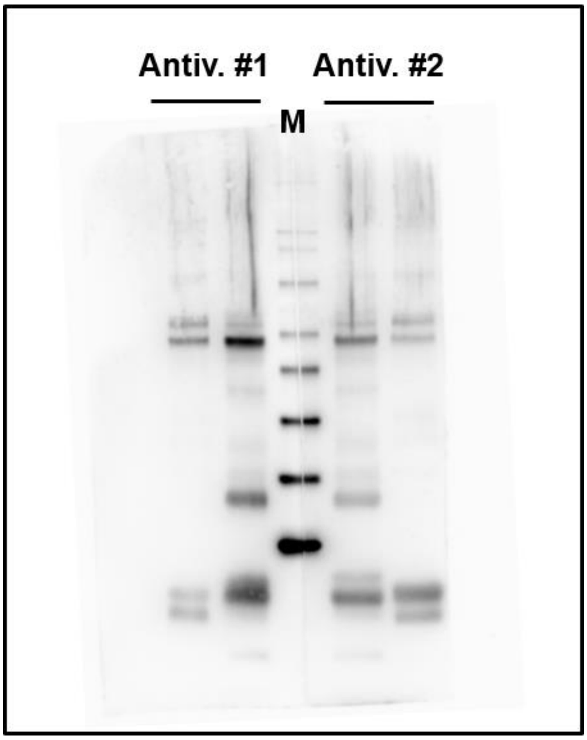

Fig S6. Non-cropped membranes of figure S5. The whole membrane was cut into two membranes with scissors after red Ponceau staining and incubated with indicated antivenoms. Please note that in Figure S5 the membrane of antivenom #2 is vertically flipped. Molecular weights of the marker are indicated in Figure S5.
